# Medical Physics Leadership Academy Journal Club (Leadership Club) program: Two‐year review in building a community of leaders

**DOI:** 10.1002/acm2.14164

**Published:** 2023-10-03

**Authors:** Ashley J. Cetnar, Kelsey Hall, Emily Y. Hirata, Dongxu Wang, Cielle E. Collins, Izabella L. Barreto, Sharbacha S. Edward, Anuj Kapadia, Michelle C. Wells, Joshua M. Wilson, Yiwen Xu, Jennifer L. Johnson

**Affiliations:** ^1^ Radiation Oncology The Ohio State University Columbus Ohio USA; ^2^ Radiation Oncology MD Anderson at Cooper Camden New Jersey USA; ^3^ Radiation Oncology University of California San Francisco San Francisco California USA; ^4^ Memorial Sloan Kettering Cancer Center New York New York USA; ^5^ University of North Carolina Chapel Hill North Carolina USA; ^6^ Department of Radiology University of Florida College of Medicine Gainesville Florida USA; ^7^ Medical Physics Washington University Saint Louis Missouri USA; ^8^ Radiology, Physics, Medical Physics Oak Ridge National Lab Oak Ridge Tennessee USA; ^9^ Piedmont Healthcare Atlanta Georgia USA; ^10^ Department of Radiology Duke University Health System Durham North Carolina USA; ^11^ Radiation Oncology BC Cancer Agency Kelowna BC Canada; ^12^ Potentia Partners Kelburn Wellington New Zealand

**Keywords:** Journal Club, leadership, virtual

## Abstract

The American Association of Physicists in Medicine began the Medical Physics Leadership Academy Journal Club in the fall of 2020. The initiative was launched to provide a forum for medical physicists to learn about leadership topics using published material, discuss and reflect on the material, and consider incorporating the discussed skills into their professional practice. This report presents the framework for the MPLA Journal Club program, describes the lessons learned over the last 2 years, summarizes the data collected from attendees, and highlights the roadmap for the program moving forward.

## 1 INTRODUCTION

Medical physicists are responsible for ensuring the safe and effective application of radiation in medical diagnosis, treatment, and research. The discipline involves developing technical and scientific expertise founded on physics, biology, epidemiology, mathematics, computer science, and medicine for the betterment of human health. Although graduate and residency programs have provided comprehensive formal training that propels medical physicists to become scientists and healthcare professionals, technical and scientific training alone is not sufficient to achieve a physicist's full potential as a leader in the field.^1^

In response, the American Association of Physicists in Medicine (AAPM) formed the Medical Physics Leadership Academy (MPLA) in 2014 and devoted the 2016 AAPM Summer School to leadership development topics. Impact International (Ambleside, UK), a consulting company specializing in leadership consulting, was brought in to conduct an assessment of the leadership and professional development skills of AAPM members. Participants of the Summer School were invited to participate in a 360‐review assessment to gather additional data about leadership from medical physicists. A total of 50 Emotional Competency Inventories^2^ (ECI) were evaluated, and the following five key skills were found to be lacking: conflict management (93% below target), initiative (90% below target), adaptability (73% below target), empathy (60% below target), and emotional self‐awareness (43% below target).^3^ A larger 2018 study from Impact International (*N* = 980 AAPM members) identified the key leadership improvement areas for AAPM's general membership, and a separate survey of residency and graduate training programs was conducted to determine training needs and required material. The leadership improvement domains identified were Personal & Interpersonal, Professional & Developmental, and Executive & Administrative.

To improve the identified focus areas, the MPLA formulated a strategy to provide impactful education, training, resources, and mentorship to the medical physics community, specifically the AAPM membership. Efforts to engage AAPM members were led by the AAPM MPLA Community Subcommittee (MPLA‐CO). One of these key initiatives is the MPLA Journal Club, which launched in 2020.^4^ This program has been rebranded as the MPLA Leadership Club as of the fall of 2022.

In medical education, a journal club is an educational meeting where individuals discuss scientific articles published in literature.^5^, ^6^, ^7^, ^8^ The journal club serves as a forum to stay apprised of new knowledge and promote awareness of current research in the respective field.^9^ It also serves as an opportunity to critique the findings and conclusions presented in the articles and explore ways of utilizing the research in practice. The goal of the MPLA Journal Club was similar. The initiative was launched to provide a forum for medical physicists to learn about leadership topics using published material, discuss and reflect on the material, and consider incorporating the discussed skills into their professional practice.

This report presents the framework for the MPLA Journal Club program, describes the lessons learned over the last 2 years, summarizes the data collected from attendees, and highlights the roadmap for the program moving forward.

## 2 THEORETICAL FRAMEWORK

The two primary theoretical frameworks which this educational program was built was adult learning theory and social constructivist theory.^10^, ^11^ Educational resources are available for adult learners to be able to be accessed and reviewed asynchronously for when is best for individual learning. The sign‐ups are volunteer‐based and left to the members to determine which topics would be aligned with their schedules and professional leadership needs. There is no additional educational credit provided for attendees, suggesting most participants are intrinsically motivated to attend to learn and grow. The materials are practical and curated to be directly applicable to their professional roles. Discussion questions are provided in advance for the autonomy of the attendees and are designed in a way that are problem‐oriented for the topic of the month and leverage the experience of participants.

The in person monthly meetings are designed to around small group discussions. The reason for this format is that the program directors believe that learning occurs in a way that is social and can go beyond the basic acquisition of knowledge when there is collaborative dialogue with others. Articulating the ideas verbally in a small group is critical for learning within social constructivism, and this venue provides a venue for all participants to share if willing. Higher levels of understanding leadership topics can be explored when interacting with members with different experiences and perspectives. Group facilitators are assigned to moderate the discussion and be a resource to guide using Vygotsky's idea of the zone of proximal development to identify where the individual learners are and ask follow‐up questions to help build knowledge in a communal setting.^11^

## 3 METHODS

In this section, we share how Journal Club sessions for each year are organized, summarize the communication plan, explain how demographic information was obtained from participants, and present the surveys given to participants after each journal club meeting. The organization of the sessions is presented by the leadership topics, program leadership structure, and the workflow for organizers on a monthly basis. For the communication plan, we discuss both the method and timing of communication. We discuss how groups were facilitated and explain how we divided the registrants into small groups. Finally, we discuss our post‐session survey, outlining the questions that were presented to participants and how we obtained demographic data from respondents.

### 3.1 Organizing journal club sessions

#### 3.1.1 Creating a list of topics and leadership structure

At the beginning of each year, the MPLA‐CO developed a list of leadership‐based topics that constituted the curriculum for the year. Table 1 shows the list of Journal Club leadership topics and schedule through 2022. Three levels of leadership are defined within the program: Program Organizer, Session Leaders, and Session Facilitators.

**TABLE 1:** MPLA Journal Club schedule for the first 2 years of the program (2020–2022).

Date	Topic	Session Leader(s)
September 2020	Influencing	Ashley Cetnar
October 2020	Conflict Management	Dongxu Wang
November 2020	Adaptability	Emily Hirata
January 2021	Initiative	Izabella Barreto
February 2021	Self‐Confidence	Joshua Wilson
March 2021	Self‐Awareness	Anuj Kapadia
April 2021	Service Orientation	Dongxu Wang
May 2021	Accurate Self‐Assessment	Emily Hirata
September 2021	Time Management/Running Meetings	Michelle Wells
October 2021	Empathy	Izabella Barreto
November 2021	Developing Others	Emily Hirata
December 2021	Emotional Self‐Control	Anuj Kapadia
January 2022	Strategic Planning/Thinking	Kelsey Hall and Dongxu Wang
February 2022	Respect for Diversity	Sharbacha Edward and Cielle Collins
March 2022	Human Resources	Emily Hirata
April 2022	Negotiation	Yiwen Xu
May 2022	Delegation	Ashley Cetnar

The Journal Club Program Organizer oversaw the operations and logistics of the program. This included announcing upcoming Journal Club sessions on the BBS forum as well as the newsletter, managing participant registrations, and supporting the Session Leaders and Facilitators. During each session, the Program Organizer managed the virtual meeting, assigned attendees to small group breakout rooms, and sent out surveys for post‐session evaluations. The Journal Club Program Organizer was Ashley Cetnar in Year 1 and Kelsey Hall in Year 2.

Session Leaders were identified for each Journal Club Session and were responsible for curating materials to supplement the group discussion, creating discussion questions to guide participant preparation, fueling a corresponding discussion web bulletin board conversation, and finally, leading the virtual Journal Club meeting. These discussion questions and topic‐based materials, which were typically in the form of freely accessible videos, lectures, or articles, were shared with the MPLA‐CO subcommittee for review at least 2 months before each Journal Club meeting. Once approved, the materials were shared with registered participants and posted to the AAPM website for access by all members. Additionally, Session Leader(s) recorded a promotional video introducing the session's material and provided some thoughts and questions to orient participants to the topic and prepare the group for discussion. Session Leaders were MPLA‐CO members in Year 1 and several additional invited Session Leaders were incorporated within Year 2.

Session Facilitators represented individuals assigned by the Program Organizer to help lead small group discussions during the Journal Club. They familiarized themselves with the material and questions, guided the discussions, and ensured everyone had opportunities to participate.

#### 3.1.2 Workflow each month for program organizer

A series of action items comprised the workflow that must be completed prior to hosting each session of the Journal Club. Table 2 lists the action items and timing of each task which is overseen by the Program Organizer to host the Journal Club session. The following includes details from the perspective of the Program Organizer starting with managing the program from the beginning of the month to the end of the month for continuity of the program.

**TABLE 2:** Summary of the “To‐Do” list for each monthly Journal Club for program organizer.

	Timing	To‐do list action items
1	Friday prior to Journal Club	Schedule facilitators and groups
2	Day of Journal Club around 10 AM	Update attendance list
3	Day of Journal Club, 1 PM	Host the Zoom session
4	Day of Journal Club, a few hours after the meeting	Send survey to attendees
5	15th of the month	Ensure information is provided to AAPM for next month's topic materials
6	One week after the Journal Club	Review the survey information and post to Trello
7	By the last week of the month before the next Journal Club	Create sign‐up sheet and post‐attendance survey for the next month's meeting
8	By last week of the month before the Journal Club	Review web page with next month's materials
9	Last Monday of the month	Send email to AAPM information services team leader to include in “What's New” email to membership
10	1st day of month	Tweet or re‐tweet to promote for the month
11	Rolling during the first 2 weeks as applications come in	Confirmation email to attendees
12	Before the next Journal Club	Schedule the Zoom meeting using AAPM website

##### Preparations prior to Journal Club session

Monthly Journal Club meetings occurred on the second Monday. Registration opened several weeks before each session and was on‐going until the day of the Journal Club. One business day before the meeting, the Program Organizer scheduled small‐group‐facilitators and attendee groups based on the approximate count of participants for the meeting.

To have the most effective discussion possible, Session Facilitators were intentionally chosen from a group of experienced members (typically from those on the subcommittee), and the discussion groups were created such that each small group had a range of career experience, age, gender, workplace, and specialty. The demographic information was obtained from the participant's AAPM membership profile. At this point, the schedule with group members was sent out to the Session Facilitators to ensure that no obvious conflicts of interest existed within their discussion group.

##### Preparations for day of Journal Club session

On the day of the scheduled Journal Club, the attendance list was updated approximately 3 h before the session began. If a registrant cancelled, this was indicated on the master‐list and individuals on the waitlist were invited. The breakout room groups were reorganized to reflect the changes. The AAPM information services team leader was emailed if additional participants needed to be added to the meeting.

The Journal Club sessions were hosted from 1:00 to 2:00 PM ET. The Journal Club Program Organizer and Session Leaders typically logged into the meeting 10 min before the session was scheduled to begin. This enabled troubleshooting potential issues prior to the meeting as well as allowed the Program Organizer to begin assigning breakout groups. Each meeting was conducted in three parts: introduction (10 min), breakout group discussions (40 min), and conclusion (10 min). The meetings began promptly a 1:00 PM, with introduction by the Session Leader. While ample prior preparation was dedicated to group organization, we found a need to dedicate this introduction time to make some last‐minute changes to accommodate unanticipated absences or unexpected guests. The Program Organizer typically made attempts to maintain a minimum of four people per breakout room, often requiring a complete last‐minute reshuffle if needed due to attendance gaps. A log of attendance and absences were maintained for the program.

A 40‐min timer was set for the small group discussions. These discussions were led by the pre‐assigned Session Facilitator in each group. The larger Journal Club reconvened for 10 min to summarize breakout room discussions. The final 5 min was reserved for closing remarks, and the Program Organizer posted links to the feedback survey as well as the signup form for next month's Journal Club in the meeting chat.

##### Post‐Journal Club session concluding tasks

One week after Journal Club, the Program Organizer reviewed the survey information, the poll results were downloaded into a spreadsheet for recording and archival by the subcommittee. The Program Organizer made note of any interesting trends and comments to address at the next MPLA community subcommittee meeting. The Program Organizer posted the results to the MPLA Community project management board for committee review. Trello (Atlassian, Sydney, Australia) was used to manage tasks, deadlines, material review, and communication for the program management. Kanban project management concepts were implemented by using columns such as “To Do”, “Doing”, and “Done” Key references and documents were also listed on the Trello board for our team.

##### Preparation for the next month's session

Finally, each Journal Club cycle was completed with advanced preparation for the following month's topic. By the 15th of the month, information was provided to AAPM for the upcoming topic materials. The Program Organizer coordinated with the upcoming Session Leader(s) to ensure their materials were sent to the AAPM website coordinators to facilitate webpage creation with the appropriate session materials.

By the last week of the month, the Program Organizer reviewed the web page to ensure that the materials and hyperlinks were accessible and accurate before release. On the last Monday of the month, the Program Organizer sent an email to the AAPM information services team leader requesting an advertisement for the upcoming session in the AAPM members e‐newsletter (What's New) distributed on the Friday of that week. Information about the upcoming journal club was also posted on the MPLA's social media feed (Twitter and Facebook). Of all methods of advertisement, the e‐newsletter was found to be the largest source of publicity for the event as shown in Figure 7.

### 3.2 Communicating about sessions

#### 3.2.1 Pre‐Journal Club communication

**FIGURE 1:**
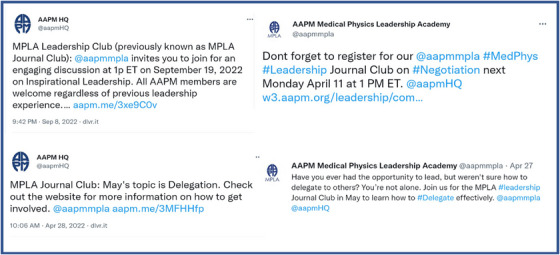
Examples of promotional tweets advertising Journal Club Session.

Each Journal Club monthly cycle began with social media posting to promote that month's Journal Club topic. This was done through Twitter where the AAPM HQ and AAPM MPLA accounts both shared the topic, date, and time of the session, shown in Figure 1 along with a link to a Google form for registration. The social media reminders were found to be a valuable registration pathway for many of the event participants, second only to the newsletter.

Event signups were accumulated using a Google registration form sent via email and/or social media posts, with the majority occurring shortly after the email or social media posts went live. However, some individuals signed‐up on the morning of Journal Club. Each participant received a confirmation message from the Program Organizer following their registration. The confirmation email was the first form of communication upon registering and provided the date, time, topic, materials, and schedule for the session. The email also contained information about cancellation which aided session planning. The template for this communication can be found in Appendices A–D. The Program Organizer monitored the number of participants over time, and if there were too many registrants for meaningful discussion in the breakout rooms, the Google form was updated to indicate that the session was full and interested members could sign‐up on a waitlist. An example of this form can be found in Appendix C.

#### 3.2.2 Post‐Journal Club communication

The final piece of communication between Journal Club leadership and session participants came in the form of a feedback survey. In the last 5 min of the Journal Club meeting, the link to the feedback survey was posted in the Zoom chat‐box, along with a link for the following month's Journal Club sign‐up page. Finally, within a few hours of the completed session, the feedback survey was also emailed to attendees. The survey content and data collected from survey responses are discussed further in the following sections.

#### 3.2.3 Follow‐up message containing survey link

Each month an additional Google form was created that served as the follow‐up survey that participants completed after the Zoom meeting. Question types included multiple choice and Likert Scale questions to provide feedback for the session. A free response section was included in the survey for general comments. Participants were asked whether they would attend another session, how they initially found out about Journal Club, whether they felt that their voice was heard when they wanted to participate, and whether they would be interested in continuing the discussion in another format after the session. In the rating from 1 to 5, participants were asked several questions about the overall structure and were asked to rate each aspect from terrible (1) to great (5). An example of the survey can be found in Appendix E.

### 3.3 Collecting demographic data

Survey respondent demographics were obtained from self‐reported data from AAPM member profiles. All Journal Club participants were AAPM members, and guests were not permitted during this initial 2‐year phase of the program. Demographic information was organized by participant by gender, AAPM membership‐type, number of years in medical physics, and medical physics practice specialty. These metrics, paired with survey responses, allowed us to organize the feedback received by the relevant characteristics of our participants.

## 4 RESULTS

### 4.1 Program registration

**FIGURE 2:**
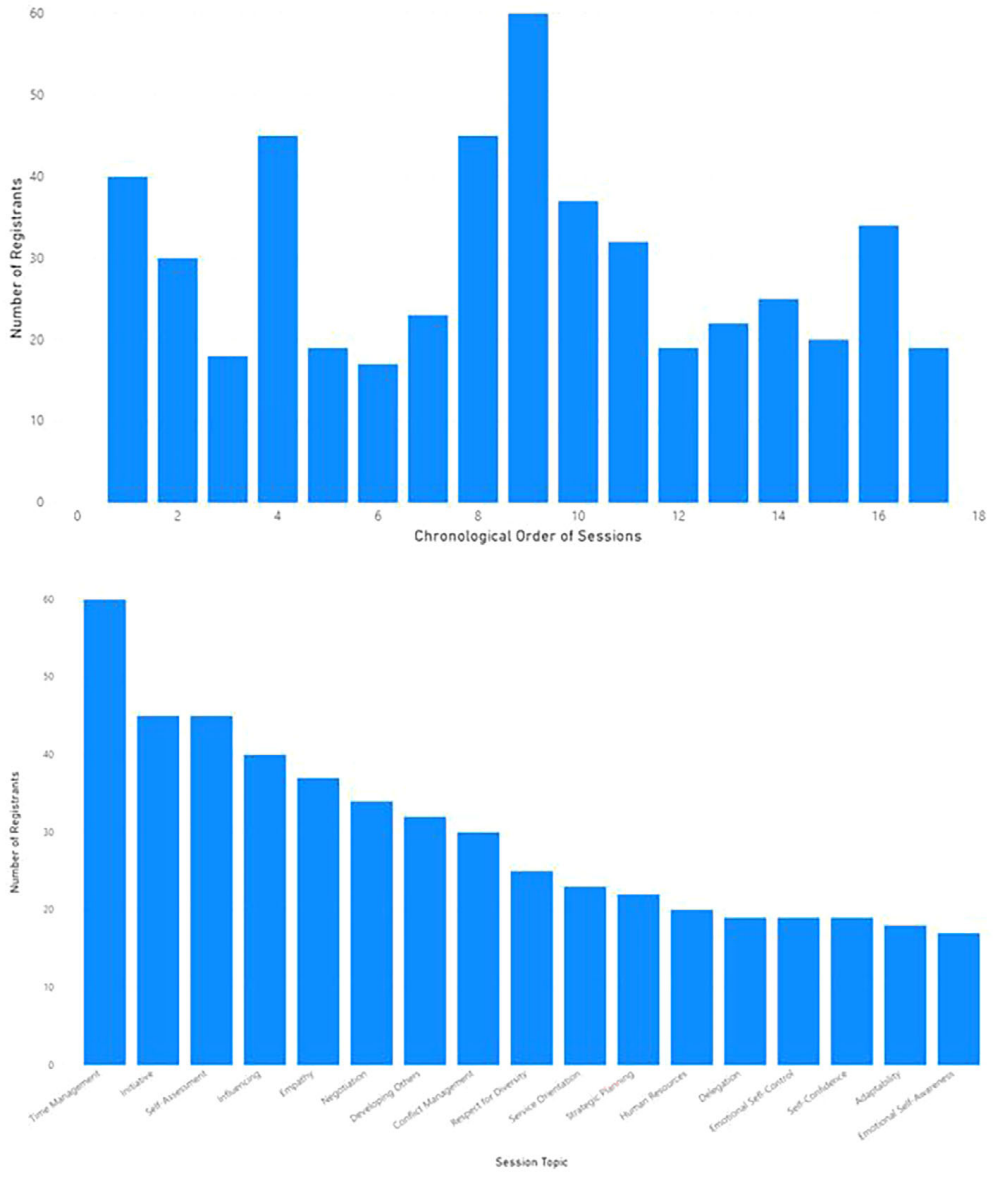
Registration for MPLA Journal Club Sessions in the first 2 years of the program by chronological order and by topic.

Overall, 172 members of the AAPM registered for at least one MPLA Journal Club session within the first 2 years of the program. The registrants by session and topic are shown in Figure 2. The average number of total registered sessions was 2.9 (STDEV 2.9) per member. Of the total 172, 10 registrants were members of the MPLA‐CO subcommittee who registered for an average of 8.1 (STDEV 5.3) sessions.

### 4.2 Demographics

**FIGURE 3:**
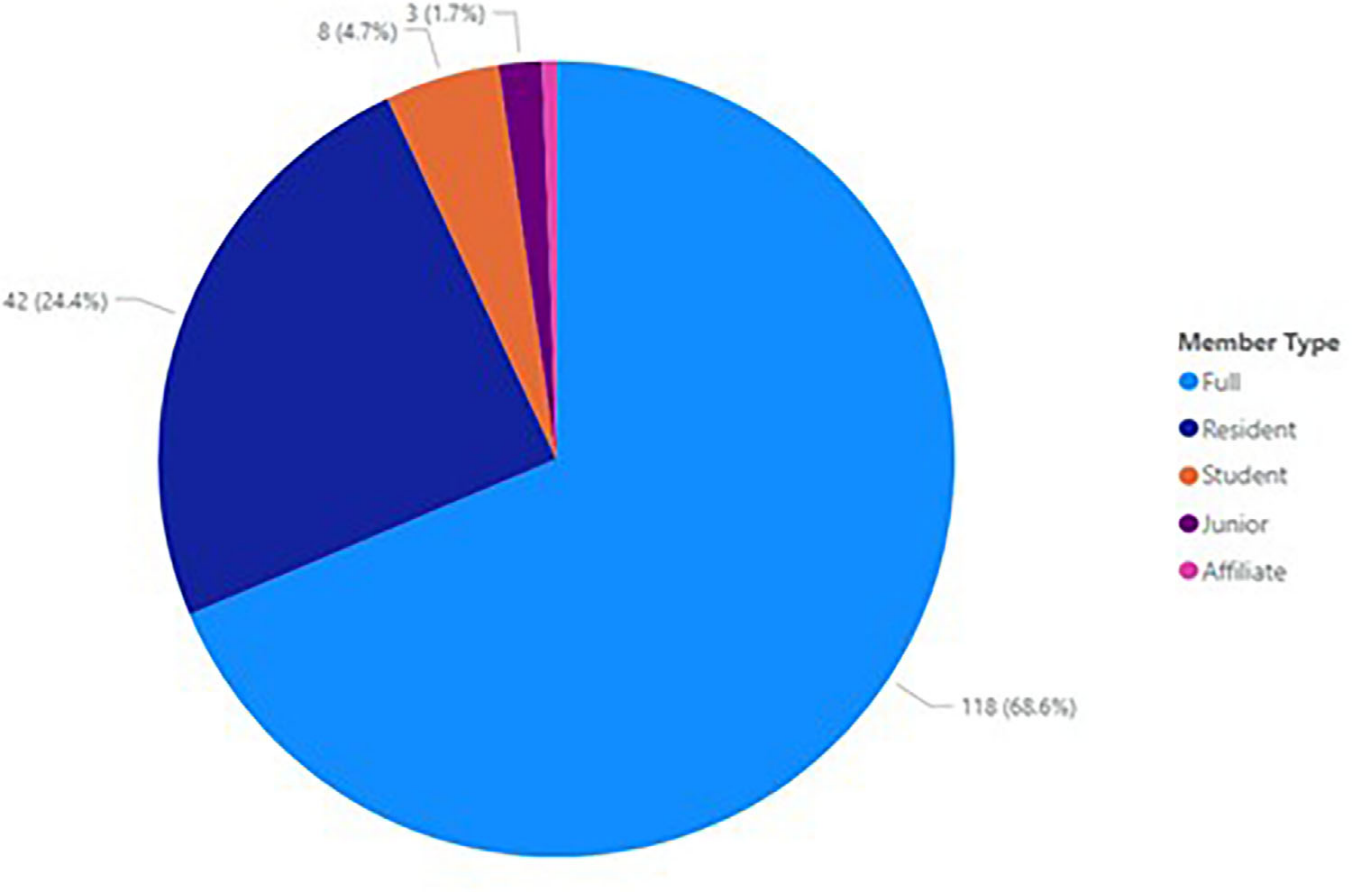
AAPM membership types of MPLA Journal Club registrants.

Demographics evaluated for registrants in the program included membership type, year when a member became a full member, specialty, and gender, based on self‐reported data from the AAPM Member Directory. Full members constituted 69% of registrants as shown in Figure 3.

**FIGURE 4:**
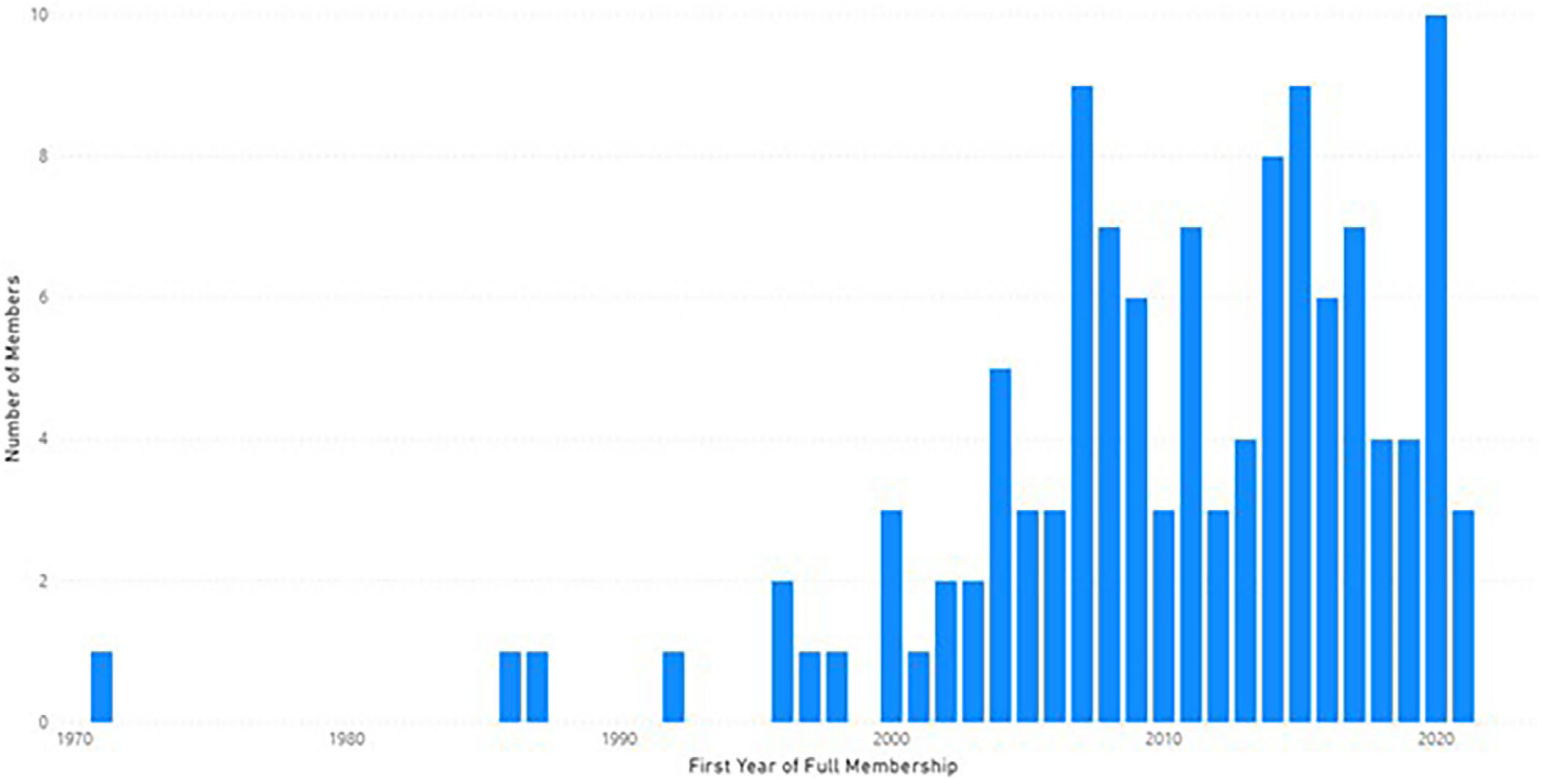
Histogram of number of MPLA Journal Club registrants by first year of full membership in the AAPM.

Of the full members, many of the registrants would be considered early career medical physicists based on when they joined the organization as full members shown in Figure 4. The next largest category was mid‐career physicists with few seasoned medical physicists registering for the program.

**FIGURE 5:**
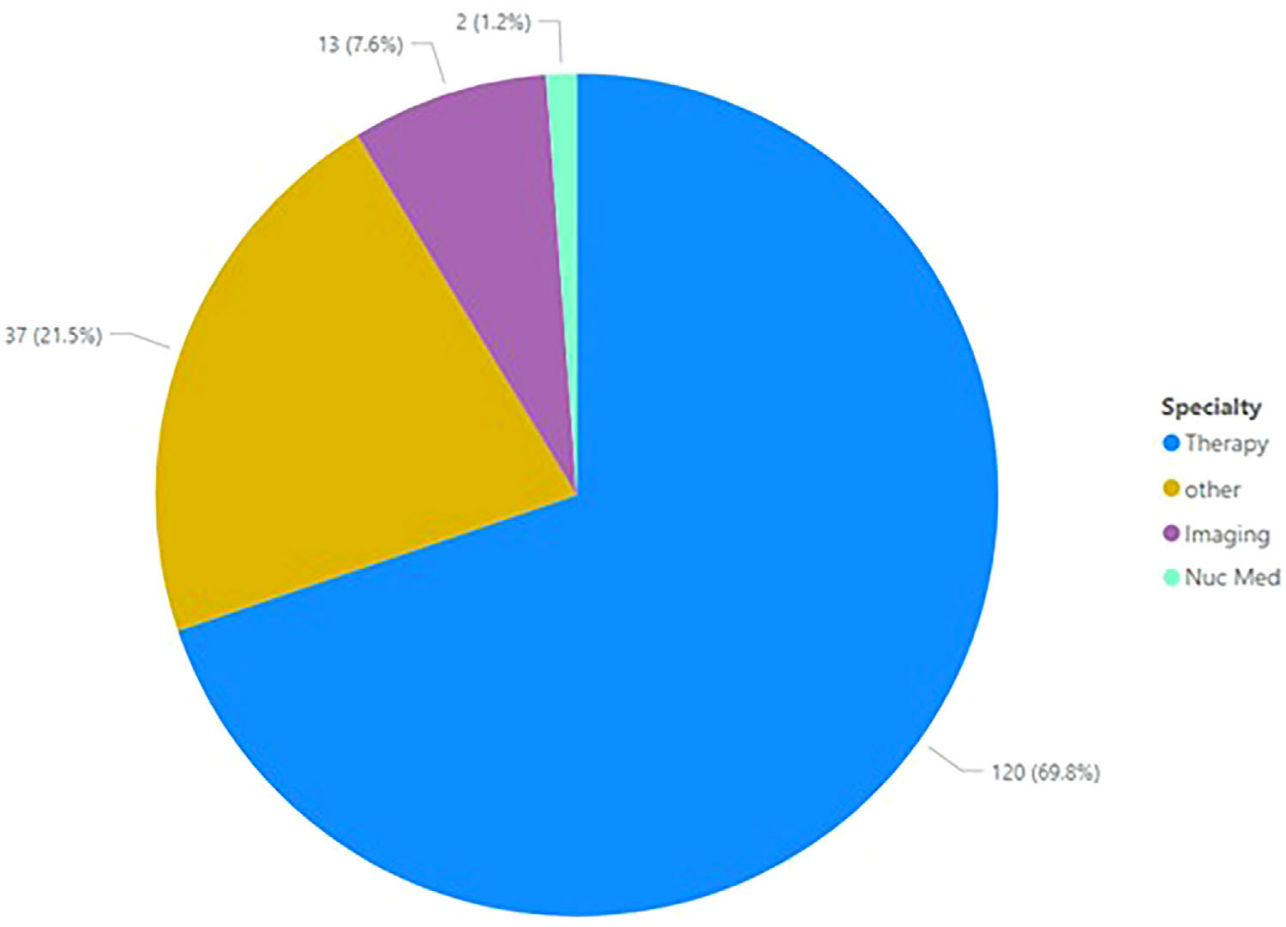
Identified primary specialty in medical physics for registrants.

Overall, 70% of registrants identified Radiation Oncology as their primary specialty denoted as “Therapy.” Radiology was denoted as “Imaging”, and Nuclear Medicine was denoted by “Nuc Med” with specialties shown for each year of the program in Figure 5. Within the database, members can select more than one specialty. If more than one specialty was designated, a participant was designated to their primary specialty (>50%). Member specialty was considered “Other” if information was not disclosed within the member's AAPM Membership Profile.

**FIGURE 6:**
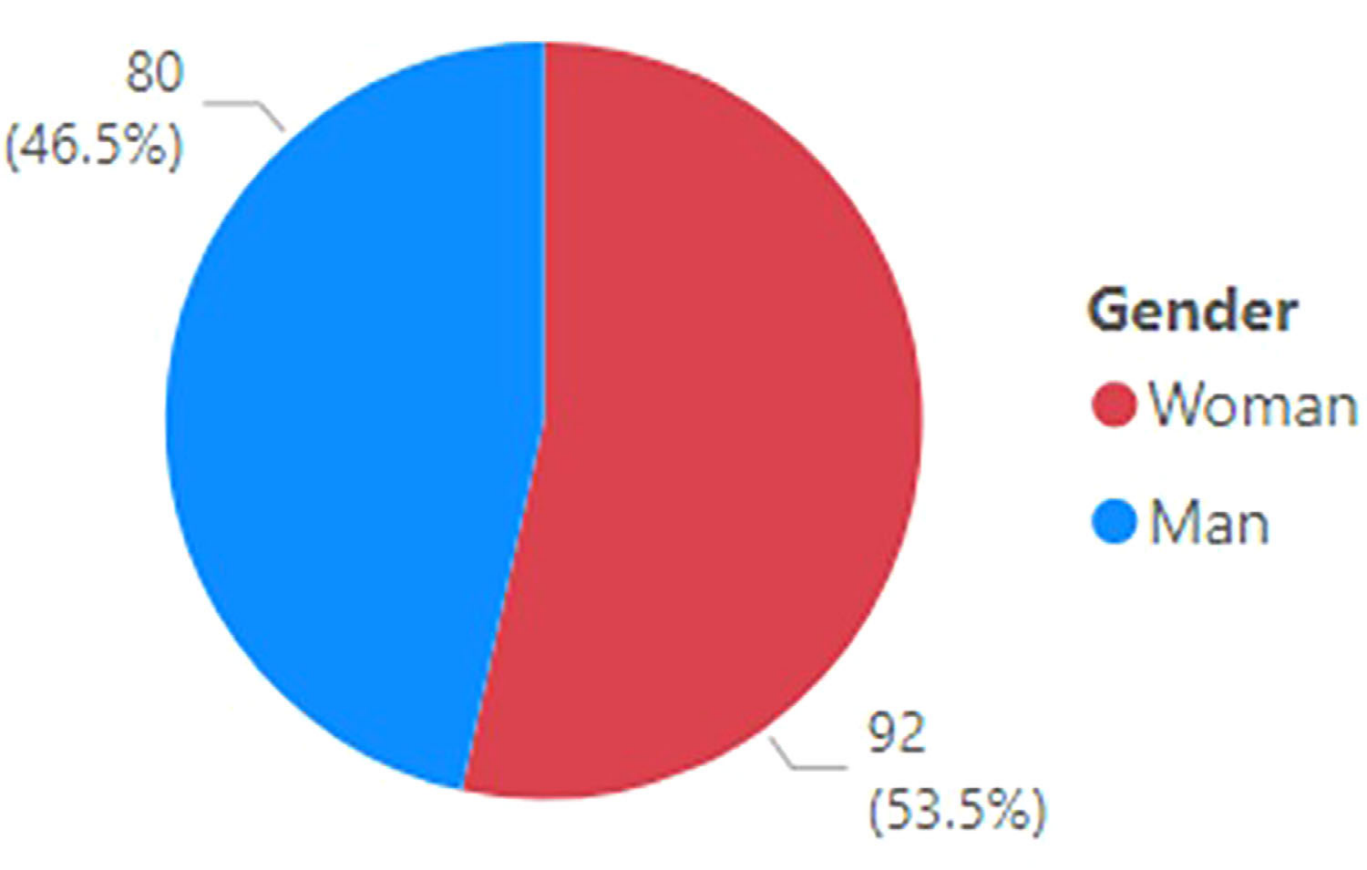
Registrants for MPLA Journal Club by gender.

Women constituted 53% of the registrants for the program based on the AAPM demographic database shown in Figure 6. This representation of women in the sessions was larger than the proportion of women within the overall organization, which is currently 23.2%. The percentage of women among the repeat attenders (i.e., attending two or more sessions) was found to be 68% in Year 1, and over 2 years this was 51% for women as repeat attenders.

### 4.3 Attendance

Registrants were categorized as “attended”, “canceled”, or “no show” based on their attendance which was manually recorded by the Program Organizer for the virtual Journal Club sessions. Overall, there were 132 unique attendees in the first 2 years of the program. Overall, 80% of the registrations resulted in attendance of the session, 8% of the registrants cancelled prior to the session, and 12% did not show up for the registered session. Since the small groups were organized and scheduled with registrants, the percentage of “no show” registrants presented logistical challenges for real‐time optimization of members within groups to foster diverse participants in small group discussions.

Between the first 2 years of the program, we identified members attending only one session and those attending more than one session. In the first year, 54% of attendees attended more than one session and this percentage increased to 69% in the second year of the program. We observe an increase in participant retention of 15% as the program has matured.

### 4.4 Survey responses

At the conclusion of each Journal Club session a survey was sent to the attendees seeking feedback on the current session as well as inputs for future meetings. Examples of these questions include:

1. Would you attend another MPLA Journal Club?
2. Did you feel that you were able to have your voice heard when you wanted to participate?
3. How did you initially find out about Journal Club?
4. On a scale from 1 to 5, how relevant and helpful do you think Journal Club was for your job?

Over the course of the first year, we received 65 survey responses. Sixty four indicated they would attend another MPLA Journal Club, one responded with “maybe”, and none of the respondents indicated they “would not attend another Journal Club”. 100% of participants indicated that they were able to have their voice heard when they wanted to participate during the Journal Club session. In Year 2, there were 66 survey respondents. 100% of responses indicated they would attend another MPLA Journal Club, and 100% indicated they were able to have their voice heard when they wanted to participate.

**FIGURE 7:**
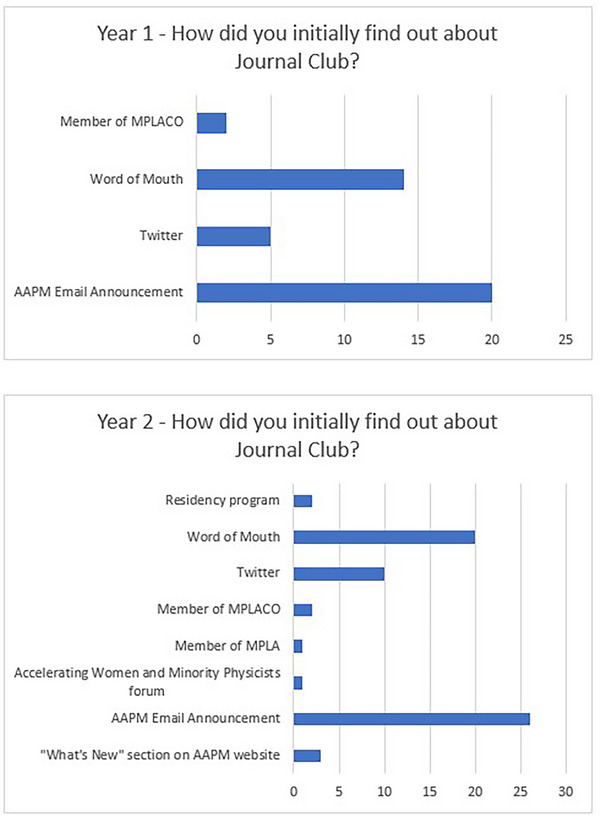
How participants found out about the MPLA Journal Club.

The survey indicated that most attendees learned about the MPLA Journal Club through the AAPM Email Announcement or learned about the program from a colleague (results shown in Figure 7).

**FIGURE 8:**
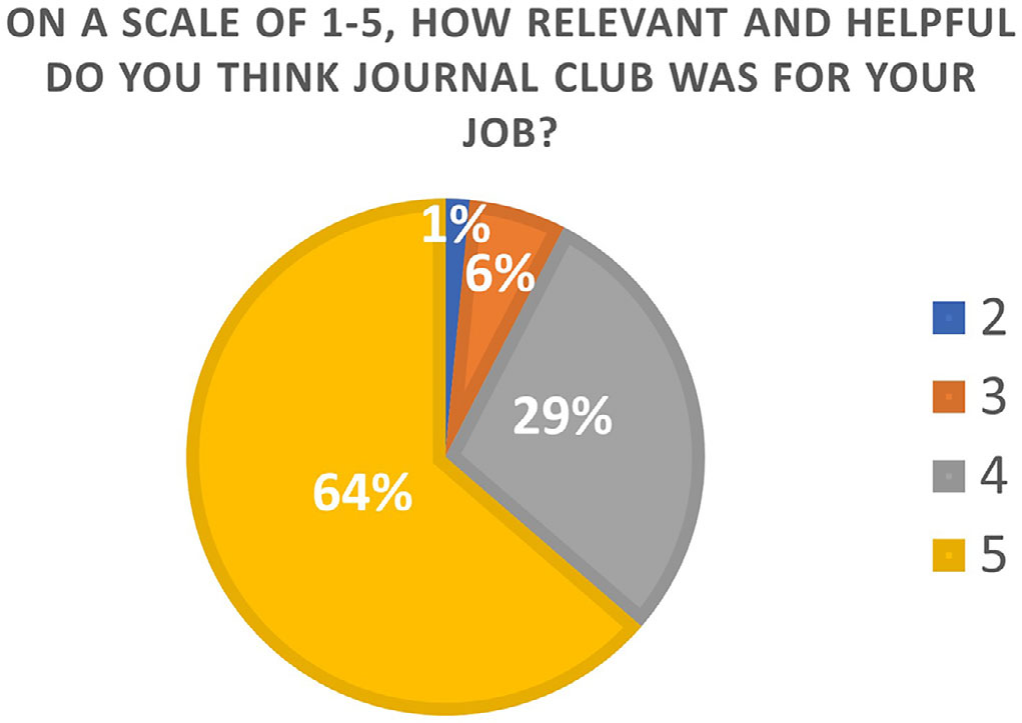
Attendee responses for relevance and helpfulness of the Journal Club session to their job. Respondents could select from Likert scale from 1 (terrible) to 5 (great). None of the participants indicated 1 in the data, so 1 is not represented on the chart.

In Year 2, an additional question was asked for participants to rank on a scale from 1 to 5 the relevance of the Journal Club to their job where 5 indicated the highest score shown in Figure 8. Within the survey there was an option for open response feedback where qualitative assessment of the program was provided. Responses were overwhelmingly positive including comments about usefulness of the content, opportunity to discuss content with diverse physicists, organization of the sessions, and overall appreciation for the program. The full list of open‐ended feedback can be reviewed in the Appendix F of this manuscript.

## 5 DISCUSSION

The background and demographics of the Journal Club attendees were shown in the results section above. In some respects, the Journal Club attendees were similar to the general AAPM membership, whereas in other aspects the attendee demographics were quite distinct. For example, with respect to gender composition, majority of journal club attendees were female, whereas the general AAPM membership is predominantly male. This is possibly correlated to the large representation of early and mid‐career physicists amongst the Journal Club attendees. The gender composition among AAPM members who joined AAPM since 2003 shows a trend toward parity to balance gender.^12^ Still, this does not fully account for the nearly 2:1 women‐to‐men ratio of the repeated attendees of this program.

Two questions in the attendee survey were helpful to reflect attendees’ overall assessment of the sessions: “Would you attend another MPLA Journal Club?” and “Is this session of value?” Every respondent reported that they would attend another session, which is compelling for articulating the value of the sessions to those willing to complete the survey. 93% of the respondents strongly agreed or agreed that the journal club content was relevant to their job. The authors interpret that the material presented in journal club meetings was representative of the needs of a typical medical physics work environment and the current format for the program was effective for discussing leadership topics relevant to medical physicists. In the future, the organizers of the Journal Club aim to develop quantifiable metrics such as pre‐ and post‐session assessments with situational questions as additional ways of understanding the impact of these sessions on the participants’ leadership development.

The topics selected in the first year of the journal club were considered “Foundation of Leadership” by the MPLA. These topics were selected based on the MPLA's needs assessments efforts, which included surveys from the 2016 AAPM Summer School, each of the leadership topics were broad, and specific resources were curated to help guide participants to learn more about multiple aspects of the subject that could be discussed in a 60‐min journal club session. While all materials were presented and approved via peer review within the MPLA subcommittee members, the organizers recognized that the selection of the reading materials, podcasts, or videos were subject to the knowledge and preferences of each session organizer and as a result they might only present a limited perspective of each topic. We anticipate that incremental refinement of topic material over future years of the program will lead to continued quality improvement in MPLA's leadership training effort.

It is worth noting that the organizers behind the Journal Club exercised great effort in avoiding conflicts‐of‐interest by attendees within their breakout groups for discussion in the virtual environment. Attendees from the same institution, especially those reporting relationships with each other, were assigned to different breakout rooms to foster open dialogue on leadership. Within each breakout room, the pre‐assigned facilitators were instructed to maintain a balanced and polite atmosphere where all attendees would have the opportunity to speak and be heard freely.

Our surveys have shown that the most valuable ways of advertising the program to members was through AAPM email and by word of mouth from participants. While this question had initially been intended to learn how the respondent initially heard of the program, the meaning could be interpreted in how the participant was informed about the most recent session as the program continued. Year 2 saw a larger proportion of residents attending journal club sessions compared to Year 1. Residency programs view the MPLA Journal Club as a complementary mechanism to meet the Commission on Accreditation of Medical Physics Education Programs (CAMPEP) guidelines on leadership, professionalism, and ethics education. An ongoing consideration is that residents may not have experienced the challenges that are often encountered throughout a longer period of time and at later stages of their career. Placement of attendees within each breakout room was considered in advance to maintain a balance in career stages across breakout room attendees to promote rich discussion. However, a balanced group is only possible if mid‐ and late‐ career physicists continue to join the discussions to share their experiences with early career physicists.

Finally, upon consideration of feedback from multiple attendees in the first 2 years, the MPLA‐CO voted to rebrand “MPLA Journal Club” to “MPLA Leadership Club” to better reflect the format for the meetings and content discussed in the sessions. Due to members having a negative preconceived notion of what a “Journal Club” would be like in a traditional sense, the committee moved to rebrand the program to encourage more members to participate. The organizers are mindful that the name “Leadership Club” may unnecessarily convey the impression of a closed elite group, which is not the intention of the rebranding. To avoid this misperception, the organizers will clarify the purpose and attendee composition in its future marketing and publicity efforts.

## 6 CONCLUSION

The MPLA Journal Club has been a strong activity conducted by the MPLA to provide leadership training to AAPM members. We plan to continue the MPLA Journal Club (now rebranded as the “MPLA Leadership Club”) for the foreseeable future. The MPLA‐CO subcommittee will continuously review the data gathered, provide recommendations for program improvement, and incorporate suggestions into the execution of the Leadership Club in the coming years of the program.

## AUTHOR CONTRIBUTIONS

Ashley J. Cetnar—Conceptualization, Methodology, Data Collection, Data Analysis, Writing—Original Draft, Writing—Review & Editing, Supervision. Kelsey Hall—Data Collection, Data Analysis, Writing—Original Draft, Writing—Review & Editing. Emily Y. Hirata, Dongxu Wang, and Cielle E. Collins—Data Analysis, Writing—Original Draft, Writing—Review & Editing. Michelle C. Wells, Izabella L. Barreto, Anuj Kapadia, Sharbacha S. Edward, Yiwen Xu, Joshua M. Wilson, and Jennifer L. Johnson—Resources, Writing—Review & Editing

## CONFLICT OF INTEREST STATEMENT

The authors declare no conflicts of interest.

## Supporting information

Supporting Information
