# Supplementary material for: Medical Physics Leadership Academy Journal Club (Leadership Club) program: Two‐year review in building a community of leaders
Source: J Appl Clin Med Phys. 2023 Oct 3;24(11):e14164. doi: 10.1002/acm2.14164 (PMC10647985; doi:10.1002/acm2.14164)

# Supplementary Material

## Appendix A – Communication Plan

- Tweet or re-tweet to promote for the month – 1st day of the month
  - Typically, MPLA marketing team AAPM HQ will create an initial Tweet and I will re-Tweet to help promote the event at the beginning of the month.
- Confirmation email to attendees – rolling during the first two weeks as applications come in
  - The Google form will accumulate sign-ups (most of them right after the email goes out to membership or the Social Media posts). However, people will still sign-up on the morning of the Journal Club. Monitor the numbers over time and if we’re getting too many registrants to facilitate, you can update the Google form to indicate we’re currently full, but members can be placed on the waitlist.
  - Template for communication can be found in Appendices D and E for reference. Please update the highlighted areas with the relevant information for the month and include an Outlook invitation with the Zoom meeting information for the invited attendees.
- Schedule facilitators and groups – Tentative schedule on Friday before Journal Club
  - We copy and paste the registration information into an excel document so that I can track the participant sign-up. Members of MPLA-CO are tagged as potential facilitators for the group. AAPM profiles are reviewed for the members so diverse groups can be created based on how long they have been members of the organization, member type, specialty, gender, workplace, etc. Group size should be ~4-5 participants in each breakout room. I will send this schedule to the facilitators to make sure there are not any obvious conflicts of interest the Friday before, but there will be adjustments based on cancellations and no-shows.
- Update attendance list – Monday of Journal Club around 10 AM
  - There will be cancellations and no-shows. If someone emails to cancel, I indicate this on the master-list to keep track of those canceling so that we can invite others in case there is a wait list and then re-organize the breakout groups.
  - Email AAPM information services team leader if any additional participants need to be added to the Zoom meeting.
- Host the Zoom session
  - On the day of the Journal Club, log into the meeting using the host account about 10 minutes before the session to help troubleshoot any potential issues that may arise. You can start assigning breakout groups as soon as participants begin arriving. You can also set a timer in the settings to 40 minutes for the discussion. After the introduction you can invite attendees to enter the breakout rooms. Try to put at least 4 people in each group and combine on the fly if necessary. Mark those who attended and no-shows for the attendance.
- In the last 5 minutes of the meeting, post links to the Feedback Survey and to next month’s Journal Club sign up in the Zoom Chat
- Send survey to attendees – within a few hours of Journal Club
  - At the conclusion of the session, send the survey to those who attended the session.
- Ensure information is provided to AAPM HQ for next month’s topic materials – 15th of the month
  - Half-way through the month, touch-base with the next month’s facilitator to insure their materials have been sent to AAPM HQ team to create the webpage with their materials.
- Review survey data – One week after the journal club review the survey information and post to Trello
  - After about a week, you can download the poll results to an excel sheet. Make note of any interesting trends to comments to specifically address at the next MPLA-CO meeting. Attach this file to the Trello card and move to the “complete” column.
- Review web page with next month’s materials – By last week of the month before the journal club
  - By the last week of the month, check and make sure the website is accessible and accurate. Check the hyperlinks and that everything would be user friendly before release.
- Send email to AAPM HQ to include in “What’s New” email to membership – Last Monday of the month
  - The last Monday of the month email AAPM HQ to remind to include in the AAPM “What’s New” email. This email will go to members on the Friday of that week. This is our biggest source of publicity for the event.

## Appendix B – Summarized To-Do List for Program Management Each Month

The recurring meeting is scheduled for the 2^nd^ Monday of the month. The following is the list of items to complete throughout the month.

- Tweet or re-tweet to promote for the month – 1st day of the month
- Confirmation email to attendees – rolling during the first two weeks as applications come in
- Schedule the Journal Club Zoom meeting using AAPM website
- Schedule facilitators and groups – Finalize the tentative schedule on Friday before Journal Club
- Update attendance list – Monday of Journal Club around 10 AM (Three hours prior to the meeting)
- Log in 10 minutes early to host the zoom session – Journal Club Monday at 1 PM ET
- Send survey to attendees – within a few hours of Journal Club
- Ensure information is provided to AAPM for next month’s topic materials by facilitators – 15th of the month
- Review data – One week after the journal club review the survey information and post to MPLA Community Subcommittee Trello Board
- Create sign-up sheet and post-attendance survey for the next month’s meeting
- Review web page with next month’s materials – By last week of the month before the journal club
- Send email to AAPM information services team leader to include in “What’s New” email to AAPM membership – Last Monday of the month

## Appendix C – Forms for Data Collection

Each month two Google forms are created.

1. Sign-up forms hyperlinks are to be included on the website and other promotional materials for members to access for sign-up.
2. Program assessment form is developed for participants to complete after the Journal Club session. This is emailed to registered participants and now available in the chat of the session for participants to complete.

The following are screen captures of the templates for reference from Year 1 Journal Club. Minor modifications were made in Year 2.


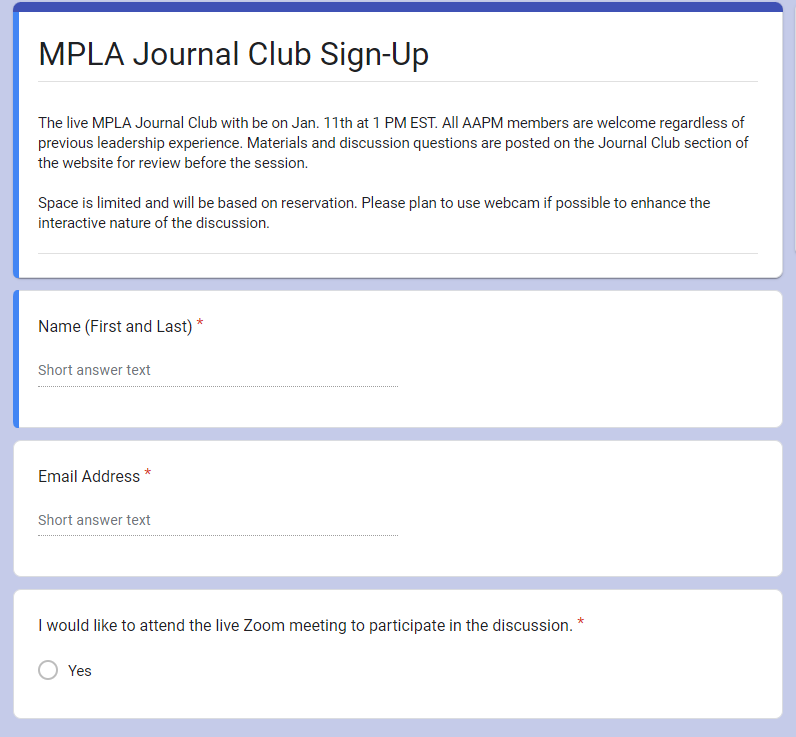


Figure 9: Example of MPLA Journal Club Sign-up


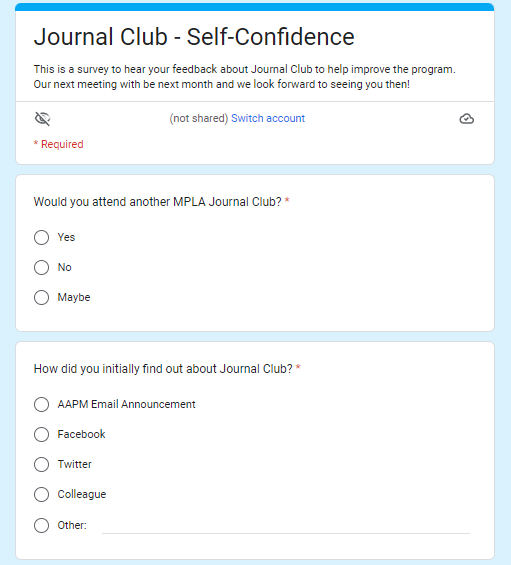


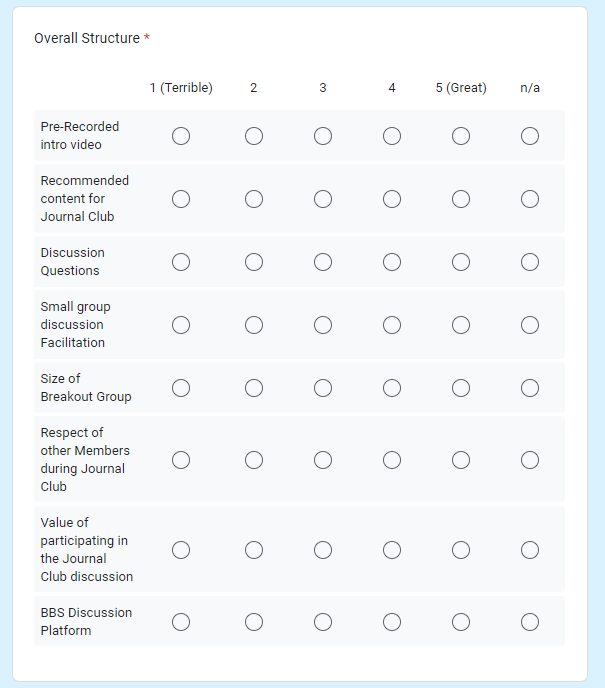


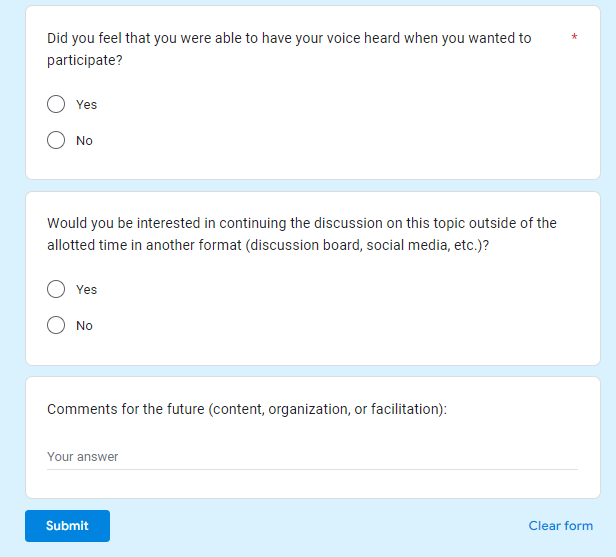


## Appendix D – Email Invitation

Email invitation sent to members who sign-up via Google form. Attach calendar invitation to the email for members to add to their calendars to save the date and time in their schedule.

Hello,

Thank you for registering for the MPLA Journal Club. We’re excited you are interested in growing as a leader and participating in this discussion.  This month’s topic is on Influencing.

Logistics:

The live MPLA Journal Club will be on Sept. 14th at 1 PM EST. The session will be hosted through Zoom and meeting details are below. Please do not share this link with others. It is strongly encouraged to use a webcam if possible for the discussion.

*insert zoom link here*

Schedule:

               1:00 EST – Brief introduction

               1:05 EST – Breakout groups

               1:45 EST – Large group discussion

Preparation:

Please review the featured material, podcast, before the session. Review the discussion questions ahead of time and think of some examples from your experience to share with your discussion group. Please also join the discussion on the BBS.

If you cannot attend:

The session is now full and there is a waitlist for participants that would like to join. If you are no longer able to attend, please let me know so that your slot can be given to someone waiting.

Please let me know if you have any questions, and we look forward to seeing you on Sept. 14th!

Thank You,

XXXX

## Appendix E - Follow-up Survey Template

Below is the template for the email communication to registrants at the conclusion of the MPLA Journal Club.

Thank you for registering for today’s Journal Club. We appreciate your engagement in the session and begin part of a great conversation about influencing. If you were able to attend today we would greatly appreciate your feedback: *insert link to Feedback Survey*

Thanks again and we hope to see you for the next conversation next month on conflict management!

Thank You,

XXXX

## Appendix F – Summary of Open Response Feedback from Participants


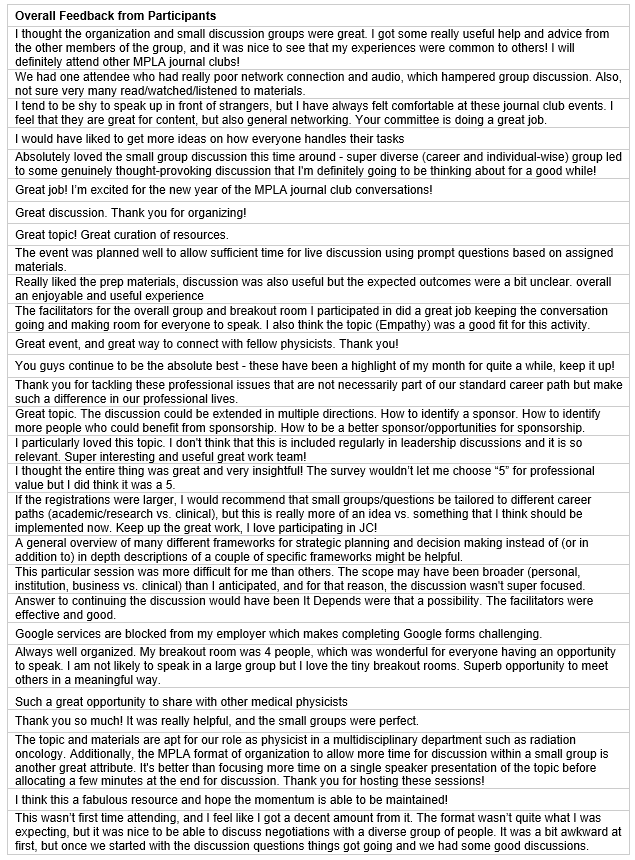

Supplement: Supplementary file 1 — Supporting Information [file ACM2-24-e14164-s001.docx]
